# Nonlinear and lagged association between ambient temperature and pre-weaning piglet mortality in Cuba

**DOI:** 10.1016/j.vas.2026.100909

**Published:** 2026-10-22

**Authors:** O. Fonseca-Rodríguez, E.A. Curbelo-Benítez, F.A. Morales-Calvo, L. Pailler-García, M.E. Licourt-Hernández, O. Uffo-Reinosa, P. Alfonso, D. de las Nieves Montano-Valle

**Affiliations:** a Unitat mixta d’Investigació IRTA-UAB en Sanitat Animal, Centre de Recerca en Sanitat Animal (CReSA), Campus de la Universitat Autònoma de Barcelona (UAB), Bellaterra, Catalonia, 08193, Spain; b IRTA. Programa de Sanitat Animal, Centre de Recerca en Sanitat Animal (CReSA), Campus de la Universitat Autònoma de Barcelona (UAB), Bellaterra, Catalonia, 08193, Spain; c Epidemiology Group, National Center for Animal and Plant Health (CENSA), World Organization for Animal Health (WOAH) Collaborating Center for the Reduction of the Risk of Disaster in Animal Health, San José de las Lajas, Cuba; d Centro Nacional de Sanidad Agropecuaria (CENSA), Laboratorio de Ensayo para el Control de la Calidad de los Alimentos (CENLAC), San José de las Lajas, Cuba

**Keywords:** Pre-weaning piglets, Heat, Cold, Case-crossover design, Distributed lag non-linear model

## Abstract

Climate change is increasing heat exposure, yet epidemiological evidence on temperature-related mortality in pre-weaning piglets remains limited, particularly in humid tropical production systems. We assessed the immediate and delayed association between daily mean ambient temperature and pre-weaning piglet mortality using records from 14 commercial farms in 12 Cuban municipalities during 2022–2024 (17,599 deaths). Farms mainly use open or semi-open naturally ventilated maternity buildings with limited active microclimate control. Municipality-level temperature and relative humidity were obtained from ERA5-Land. We applied a space–time-stratified case-crossover design with conditional quasi-Poisson regression and a distributed lag non-linear model over lags 0–7 days, adjusting for relative humidity. The cumulative temperature–mortality association was non-linear and strongly heat-dominated, with a model-derived minimum-mortality temperature (MMT) of 23.7 °C; the increase at the extreme cold tail was less precise. Effects were predominantly acute. At lag 0, the estimated RRs were elevated at both the maximum observed temperature of 31.2 °C (RR 1.49, 95% CI 1.06–2.09) and the minimum of 14.3 °C (RR 2.26, 95% CI 1.27–4.02); however, cold effects attenuated rapidly and contributed little to the overall burden. The estimated temperature-attributable fraction was 16.6%, with most of the burden associated with temperatures above the MMT (15.5%). Most heat-attributable deaths occurred on non-extreme warm days above the minimum-mortality temperature. Exposure to temperatures at or above the 99th percentile (P99) of the farm-day temperature distribution accounted for 0.7%. These findings support routine heat-risk management in farrowing environments to help reduce pre-weaning mortality and strengthen climate resilience in tropical swine systems.

## Highlights

• The temperature–mortality curve was U-shaped, with a minimum at 23.7 °C.
• The temperature–mortality association was predominantly acute at both extremes.
• The attributable burden was substantial and almost entirely heat-driven.
• Most heat-attributable deaths occurred on non-extreme warm days.
• Routine heat-risk management could reduce pre-weaning piglet deaths.

## Implications

The estimated temperature-attributable burden in Cuban commercial farms was predominantly heat-related, and most of this heat-attributable burden occurred on non-extreme warm days above the MMT rather than only during rare hot extremes.

Literature-supported, feasible heat-mitigation options for farrowing environments include improved ventilation, increased air movement, water access, and targeted cooling during the warmest hours. The present findings can inform producers, veterinarians, and animal-health authorities when prioritizing heat-risk management and climate-resilience actions in tropical swine systems.

## Introduction

Climate change is shifting the distribution of ambient temperature and modifying the frequency and intensity of temperature extremes, with important consequences for biological systems and food production (IPCC, 2021, 2023). These climatic changes constitute a major challenge for animal production because departures from thermal comfort—toward both unusually hot and unusually cold conditions—can impair welfare, reduce productivity, and increase the probability of adverse health outcomes, including mortality (Cheng, McCarl & Fei, 2022).

Newborn piglets are particularly vulnerable to cold because of their immature thermoregulation, high surface-area-to-body-mass ratio, poor insulation, and limited energy reserves. Consequently, cold exposure can rapidly cause hypothermia; the resulting weakness and reduced locomotion may impair colostrum intake and increase the risk of starvation and crushing (Gómez-Prado et al., 2022; Herpin, Damon & Le Dividich, 2002). Although cold is the predominant thermal challenge immediately after birth, high ambient temperatures, particularly when combined with high humidity, can also constrain evaporative heat loss and increase thermal strain in pigs (Godyn et al., 2020; Ross et al., 2015).

High temperatures may also influence pre-weaning piglet outcomes indirectly through the lactating sow. Heat stress in lactating sows reduces voluntary feed intake and milk production and impairs litter growth (Black et al., 1993; Muns et al., 2016; Quiniou & Noblet, 1999; Renaudeau & Noblet, 2001). Exposure to elevated temperature around farrowing can also alter sow postural behavior and reduce piglet weight at weaning (Muns et al., 2016). Together, these direct and sow-mediated pathways provide biological plausibility for a temperature–mortality association during the pre-weaning period.

Cuba offers a particularly informative setting for investigating temperature–piglet mortality associations. The country has a tropical, seasonally humid climate with maritime influence (Alonso & Clark, 2015; Bultó et al., 2006). Moreover, in Cuba, pig production frequently relies on open or semi-open maternity buildings with natural ventilation and limited capacity for active microclimate control. Under these conditions, outdoor weather may strongly influence the farrowing and lactation environment and the thermal conditions experienced by the sow–piglet dyad. This strengthens the rationale for evaluating ambient temperature as an exposure metric relevant to piglet survival, including potential risks associated with both heat and episodic cold conditions (Herpin, Damon & Le Dividich, 2002; Ross et al., 2015).

Despite this biological plausibility, field evidence linking thermal conditions to pre-weaning piglet mortality remains limited. Previous studies have evaluated temperature or local housing climate in relation to piglet survival in temperate indoor and organic outdoor systems (Schild et al., 2019; Wegner et al., 2014). However, evidence remains scarce regarding short-term non-linear and delayed temperature–mortality associations in tropical commercial production systems and the corresponding mortality burden relative to a model-derived reference temperature.

The methodological contribution of the present study lies in combining a space–time-stratified case-crossover design and a distributed lag non-linear model (DLNM) for multi-farm daily pre-weaning death counts in a humid tropical setting. The DLNM characterizes short-term non-linear and delayed associations, whereas conditioning on farm, year, month, and day of the week controls by design for time-invariant farm characteristics, long-term trends, month-level seasonality, and day-of-week patterns (Gasparrini, Armstrong & Kenward, 2010; Tobias, Kim & Madaniyazi, 2024; Wu, Li & Guo, 2021).

Accordingly, the primary objective was to characterize the short-term non-linear and lagged association between municipality-level daily mean ambient temperature and daily counts of recorded all-cause pre-weaning piglet deaths in 14 commercial farms across 12 Cuban municipalities. The secondary objectives were to (i) estimate the model-derived minimum-mortality temperature and (ii) estimate the fractions and numbers of deaths statistically attributable to temperatures below and above this reference.

## Material and methods

### Study setting

Cuba is a Caribbean archipelago with a tropical, seasonally humid climate and strong maritime influence (Alonso & Clark, 2015; Hernández, Martínez & Marzo, 2015). The climate is characterized by a rainy season typically spanning May–October and a relatively drier season from November–April (Bultó et al., 2006). Under the Köppen–Geiger climate classification, Cuba is predominantly classified as tropical savanna (Aw) (Beck et al., 2018; Peel, Finlayson & McMahon, 2007).

Mean annual air temperature ranges from approximately 24 °C in mountainous areas to 26 °C in the plains; mean maximum and minimum temperatures range from 27 to 32 °C and from 17 to 23 °C, respectively; and mean annual precipitation is approximately 1335 mm (Planos & Gutiérrez, 2020) and national climate variability is strongly influenced by extremes such as droughts (Stojanovic et al., 2025), heavy rainfall events (Alvarez-Socorro et al., 2021), and tropical cyclones (Fernández–Alvarez et al., 2020). Recent climatological analyses also report an increasing frequency of heat-wave conditions across Cuban regions (Pérez-Alarcón et al., 2024).

### Data sources and outcomes

Daily piglet mortality records were obtained from routine administrative datasets provided by the Cuban Porcine Genetic Company (GENSPOR, in Spanish: Empresa Genética Porcina) for 14 commercial genetic breeding farms located in 12 municipalities across western, central, and eastern Cuba during 2022–2024. All participating farms belonged to GENSPOR and had broadly similar management and routine mortality-recording practices. These farms were dedicated to sow reproduction, genetic selection, and piglet production up to weaning. Across farms, piglets were typically weaned at 26–33 days of age.

Farrowing accommodation consisted of open or semi-open maternity buildings with natural ventilation and standardized Flat-Deck farrowing crates/cubicles. Sows were confined in crates of approximately 1.60 × 2.40 m during farrowing and lactation. The sow and litter remained in the same unit until weaning; suckling piglets were not housed in a separate room, although the Flat-Deck system included a designated resting area for piglets. Buildings were equipped with protective curtains, suggesting limited capacity for active microclimate control and a close relationship between outdoor weather and the maternity-room environment.

The available farm-size indicator in the administrative mortality dataset was not disaggregated by production category and therefore could not be interpreted as the number of breeding sows or as the exact daily number of suckling piglets alive. Accordingly, it was used only to describe the production context and was not included as an offset in the statistical model. Daily piglet deaths were retrieved from farm epidemiological files maintained as part of routine production and animal-health monitoring, recorded by farm technical staff involved in reproduction and maternity management, and subsequently consolidated in the administrative mortality database used for this study. Records classified as removals were excluded *a priori*, and only deaths were retained, yielding daily farm-level counts of recorded all-cause pre-weaning piglet deaths. During data preparation, multiple records were aggregated into one farm–day observation, and the constructed analytical panel was checked to ensure one observation per farm–date and a continuous date sequence within each farm.

Daily mean 2-m air temperature and 2-m dew-point temperature were obtained from the Copernicus Climate Change Service ERA5-Land hourly reanalysis, accessed via the Climate Data Store (Muñoz Sabater, 2019). The corresponding ERA5-Land variables were *2m_temperature* and *2m_dewpoint_temperature*, respectively. ERA5-Land is distributed on a regular latitude–longitude grid of 0.1° × 0.1° (native resolution ∼9 km). Because precise farm coordinates were unavailable, exposures were assigned at the municipality level. Daily-mean fields derived from the hourly product were spatially averaged across grid cells within each municipality polygon and converted from Kelvin to degrees Celsius. Relative humidity (RH; %) was subsequently calculated at the municipality-day level from the spatially averaged daily mean air temperature (T) and dew-point temperature (Td), both expressed in degrees Celsius, using the August–Roche–Magnus equation (Alduchov & Eskridge, 1996):

RH = 100 × exp{[17.625 × Td/(243.04 + Td)] − [17.625 × T/(243.04 + T)]}

This procedure generated municipality-day series of daily mean temperature and relative humidity for the epidemiological analyses. Each farm was linked, according to its recorded municipality and date, to the corresponding municipality-day temperature and relative-humidity series; farms located within the same municipality consequently shared the same exposure series. The scientific description and evaluation of ERA5-Land are provided by Muñoz-Sabater et al. (2021).

### Study design and statistical analysis

We used a space–time stratified case-crossover design, implemented through conditional quasi-Poisson regression, to evaluate the association between daily mean temperature and piglet mortality (<4 weeks) in commercial swine farms in Cuba. Conditioning strata were defined by the cross-classification of Farm × Year × Month × Day-of-week (DOW), thereby controlling by design for time-invariant farm characteristics, month-level seasonality, day-of-week patterns, and longer-term temporal trends (Armstrong, Gasparrini & Tobias, 2014; Tobias, Kim & Madaniyazi, 2024; Wu, Li & Guo, 2021). In practical terms, this design compares mortality counts across days within the same farm, calendar year, month, and day of the week. Thus, the temperature–mortality association is estimated from short-term temperature contrasts between comparable days, rather than from broad seasonal or between-farm differences. This conditioning structure directly supports the objective of estimating short-term and lagged temperature-related associations while reducing confounding by seasonality, long-term temporal trends, day-of-week patterns, and time-invariant farm-level characteristics.

Short-term, potentially delayed, and non-linear temperature effects were modeled using a distributed lag non-linear model (DLNM) framework (Gasparrini, Armstrong & Kenward, 2010, 2011). Let $Y_{it}$​ denote the daily count of piglet deaths in farm i on day t. We fitted conditional quasi-Poisson models with a log link:

$$Y_{it}∼\text{quasi}-\text{Poisson}\left(\mu_{it}\right),$$

$$\text{log}\left(\mu_{it}\right)=cb\left(Tmean_{it},l\right)+ns\left(Rhum_{it},df=3\right)+\alpha_{stratum\left(i,t\right)},$$

where $cb(Tmean_{it},l)$ is a DLNM cross-basis comprising (i) a natural spline for the exposure–response relationship (2 degrees of freedom) and (ii) a natural spline for the lag–response relationship with two interior knots equally spaced on the log-lag scale, with lags l=0,…,7. Relative humidity was included as a potential time-varying confounder and modeled using a non-lagged natural spline with 3 degrees of freedom. $\alpha_{stratum(i,t)}$denotes a fixed effect for each Farm × Year × Month × Day-of-week stratum. Strata in which the sum of recorded piglet deaths across all farm-days was zero were excluded because they provided no information for estimating within-stratum temperature–mortality associations. The cross-classification generated 3528 conditioning strata, of which 1102 (31.2%) were excluded, leaving 2426 strata eligible for analysis. These retained strata comprised 10,574 of the 15,344 available farm-day observations (68.9%). After applying the 0–7-day lag structure and complete-data requirements, the final fitted model included 10,512 farm-day observations (68.5%).

The minimum mortality temperature (MMT) was identified as the temperature associated with the minimum cumulative risk within the observed temperature range; temperature effects were summarized as cumulative relative risks over lags 0–7 centered at the MMT. The uncertainty around the MMT was estimated using the approximate parametric bootstrap method proposed by Tobías, Armstrong and Gasparrini (2017), which provides confidence intervals and standard errors derived from spline-based temperature–mortality curves.

We estimated attributable fractions (AF) and attributable numbers (AN) for overall temperature following Gasparrini and Leone (2014), and further decomposed the burden using backward attribution within the DLNM framework. Heat-attributable mortality was defined as the model-estimated burden associated with temperatures above the fitted MMT, whereas cold-attributable mortality was defined analogously for temperatures below it. To characterize tail risks, we additionally computed AF and AN for extreme heat (≥99th percentile) and extreme cold (≤1st percentile), defined from the empirical distribution of daily mean temperature across farm-day observations during the study period. Uncertainty was quantified using Monte Carlo simulation (nsim = 10,000) by sampling from the multivariate normal distribution of the estimated coefficients based on their variance–covariance matrix, yielding empirical 95% confidence intervals.

For the primary AF and AN intervals, the model-derived MMT of 23.7 °C was held fixed while the cross-basis coefficients were simulated; these intervals were therefore conditional on the estimated MMT. In a sensitivity analysis, the MMT was re-estimated for each coefficient draw, and the same draw and corresponding MMT were used to recalculate the overall, heat-related, and cold-related AF and AN (Supplementary Table S3).

The maximum lag for the main model was set to 7 days, balancing biological plausibility, interpretability, and sensitivity analyses. This window was considered more appropriate for pre-weaning piglet mortality than longer lag periods, given the rapidly changing composition of the at-risk population and the predominantly acute lag pattern observed in preliminary analyses. Within this 7-day lag window, we conducted a grid search over a prespecified set of smoothing parameters. Specifically, we evaluated models with 2–4 degrees of freedom for the exposure–response function, 2–4 interior knots for the lag–response function, and 2–4 degrees of freedom for the spline adjustment of relative humidity. Candidate smoothing specifications were compared using the quasi-Akaike information criterion (qAIC), computed from the quasi-Poisson likelihood with an estimated dispersion parameter. When differences were negligible (ΔqAIC < 2), we selected the more parsimonious parameterization (Supplementary Table S2).

Sensitivity analyses refitted the selected model using maximum lags of 3, 5, 10, 14, and 21 days while retaining the same natural-spline framework. We compared the estimated MMT, cumulative relative risks at selected temperatures, overall, heat-, and cold-attributable fractions, cumulative exposure–response curves, and lag-response patterns with those obtained from the main 7-day model. Because the daily birth records required to reconstruct the population at risk were available only for 2024, we conducted a separate paired sensitivity analysis restricted to that year. We compared otherwise identical models with and without the logarithm of the reconstructed daily population of suckling piglets included as an offset; full reconstruction procedures are provided in the Supplementary Material.

Analyses were performed in R (R Core Team, 2024) using packages *dlnm, gnm, and FluMoDL*, with supporting packages for data curation and reporting (*tidyverse, gtsummary, flextable*).

## Results

Across 15,344 farm–day observations the mean number of recorded piglet deaths was 1.15 per farm–day (±2.32; range 0–63). Mean daily temperature averaged 25.8 °C (±2.3; range 14.3–31.2 °C), and relative humidity averaged 76% (±7%; range 52–95%). Over the three-year study period, 17,599 piglet deaths were recorded.

Fig. 1 summarizes the geographic coverage of the study, showing the distribution of the 14 commercial farms across 12 municipalities spanning west to east Cuba.

**Fig. 1:**
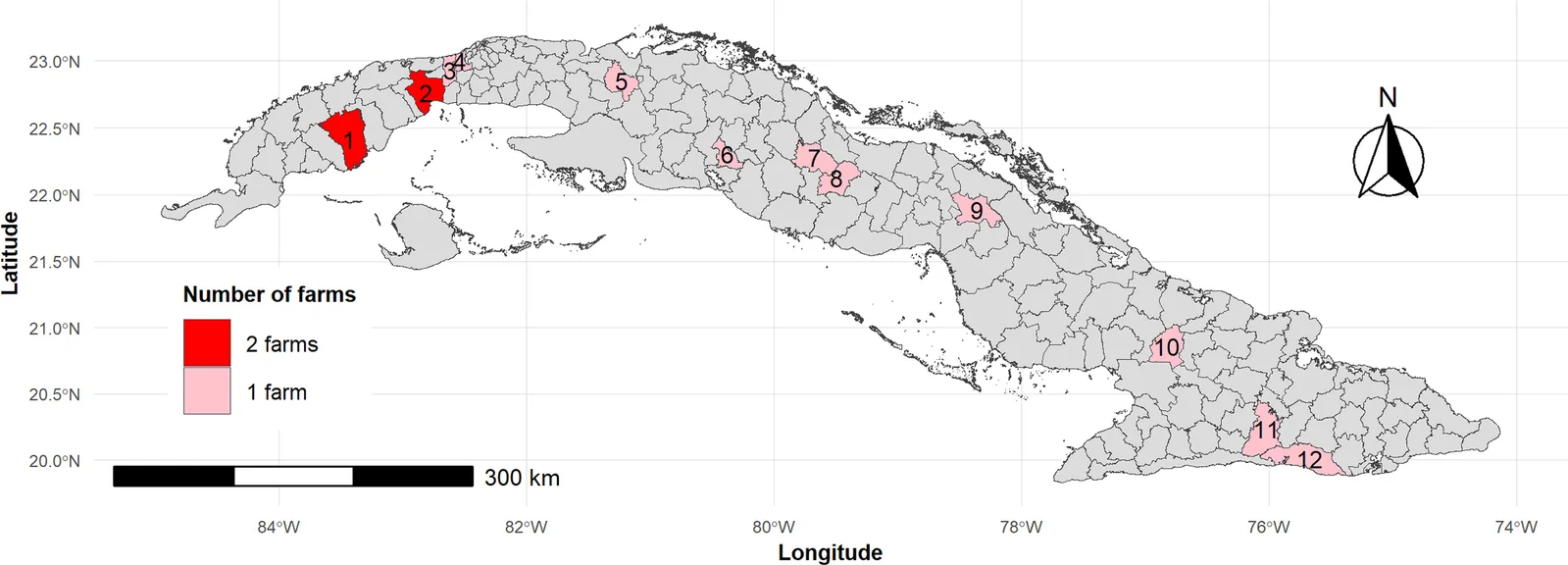
Spatial distribution of study farms across Cuban municipalities. Numbers label municipalities with study farms in west–to–east order as follows: 1—Consolación del Sur; 2—Artemisa; 3—Caimito; 4—Bauta; 5—Jovellanos; 6—Palmira; 7—Placetas; 8—Cabaiguán; 9—Primero de Enero; 10—Majibacoa; 11—Palma Soriano; 12—Santiago de Cuba.

Fig. 2 presents municipality-stratified time series of daily piglet mortality, mean temperature, and relative humidity, highlighting heterogeneity in both exposure profiles and mortality intensity across the 12 municipalities.

**Fig. 2:**
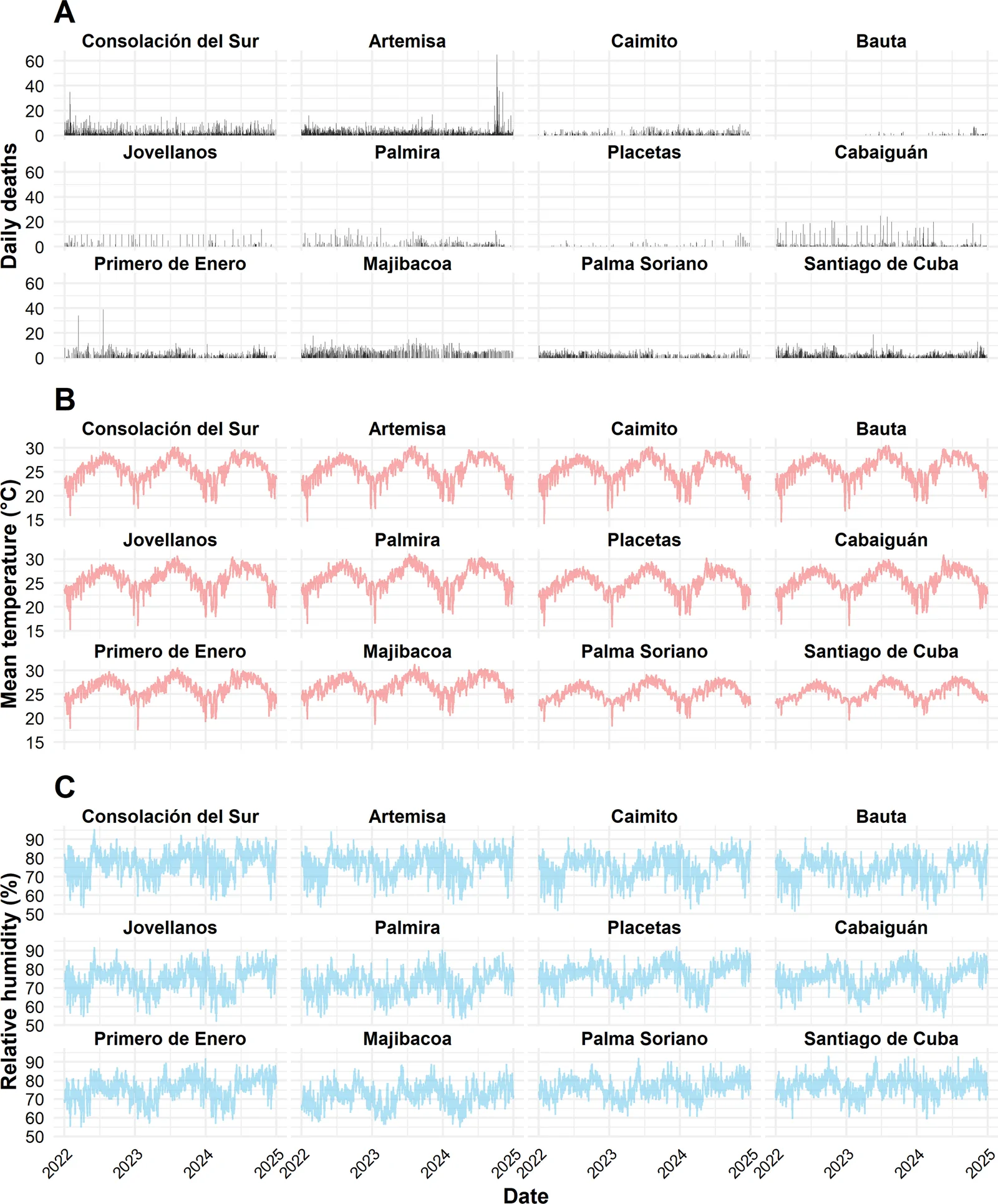
Time series of daily piglet mortality (A, black bars), mean temperature (B, coral lines), and mean relative humidity (C, blue lines), stratified by municipality.

Table 1 presents municipality-specific summaries based on municipality-day observations; the Overall row summarizes calendar-day values aggregated across all farms. Across the 12 municipalities, mean daily piglet deaths varied widely from 0.1 (SD 0.6; range 0–7) in Bauta and 0.2 (0.8; 0–11) in Placetas to 2.4 (3.2; 0–18) in Majibacoa and 3.0 (4.0; 0–65) in Artemisa. Average daily temperature ranged from 25.2 °C (2.3; 15.9–30.2) in Placetas to 26.9 °C (1.9; 18.8–31.2) in Majibacoa, while mean relative humidity varied modestly across municipalities, with means clustering around 72–77% (SD ≈5–7) and daily values spanned 52–95%.

**Table 1:** Descriptive characteristics of study farms and ambient weather by municipality, Cuba, 2022–2024.

**Municipality**	**Number of farms**	**Farm population indicator**	**Daily pre-weaning deaths**	**Mean temperature** ( °C)	**Relative humidity (%)**
Artemisa	2	5783 (826) [3412–6915]	3.0 (4.0) [0–65]	25.6 (2.4) [14.7–30.4]	76.8 (6.6) [53.8–94.0]
Bauta	1	1925 (229) [1223–2317]	0.1 (0.6) [0–7]	25.8 (2.4) [14.6–30.5]	74.3 (6.7) [51.7–90.9]
Cabaiguán	1	4260 (743) [1814–5369]	1.0 (2.9) [0–25]	25.6 (2.3) [16.2–30.8]	75.1 (6.4) [54.1–90.1]
Caimito	1	2499 (576) [1083–3393]	0.9 (1.5) [0–9]	25.6 (2.4) [14.3–30.2]	75.2 (6.9) [52.1–91.1]
Consolación del Sur	2	6520 (732) [4217–7783]	2.5 (3.0) [0–35]	25.6 (2.4) [15.9–30.1]	77.3 (6.8) [53.7–95.1]
Jovellanos	1	2912 (559) [1273–3683]	0.4 (1.7) [0–14]	25.8 (2.4) [15.4–30.6]	74.5 (6.7) [52.3–91.6]
Majibacoa	1	2964 (591) [1685–3937]	2.4 (3.2) [0–18]	26.9 (1.9) [18.8–31.2]	72.0 (6.2) [55.2–88.7]
Palma Soriano	1	2606 (471) [1330–3367]	1.3 (1.8) [0–10]	25.4 (1.7) [18.4–29.1]	76.1 (5.2) [60.8–91.8]
Palmira	1	2485 (376) [1692–3411]	0.7 (1.9) [0–15]	26.3 (2.4) [16.6–31.0]	72.1 (6.5) [53.3–90.1]
Placetas	1	3340 (245) [2492–4095]	0.1 (0.8) [0–11]	25.2 (2.3) [15.9–30.2]	76.8 (6.5) [57.2–92.0]
Primero de Enero	1	2649 (457) [1501–3489]	1.6 (2.6) [0–39]	26.3 (2.1) [17.6–30.5]	75.4 (6.4) [55.3–91.5]
Santiago de Cuba	1	2606 (451) [1370–3523]	2.1 (2.4) [0–19]	25.5 (1.6) [19.7–29.0]	77.5 (5.2) [59.5–93.0]
**Overall**	**14**	**40,548 (5369) [23,882–48,847]**	**16.1 (9.9) [0–87]**	**25.8 (2.2) [16.6–29.9]**	**75.5 (5.3) [58.4–87.4]**

Values are mean (SD) [min–max] of daily summaries. Municipality rows summarize municipality-day values; where multiple farms were present, deaths and the farm population indicator were summed and weather variables were averaged. The Overall row summarizes calendar-day totals across all farms for deaths and the farm population indicator, and calendar-day averages across farm records for temperature and relative humidity.

Fig. 3 shows the cumulative exposure–response function over lags 0–7 days. The fitted curve was U-shaped but strongly heat-dominated, with a more precisely estimated heat component than the cold component, and was centered at the model-derived minimum-mortality temperature (MMT) of 23.7 °C (95% CI: 21.9–24.6). The MMT is a model-derived reference point for the exposure–response curve; its confidence interval indicates uncertainty around the exact location of the minimum-risk point. It should therefore be interpreted on the ambient temperature scale used in this study, rather than as a precise pen-level thermal threshold.

**Fig. 3:**
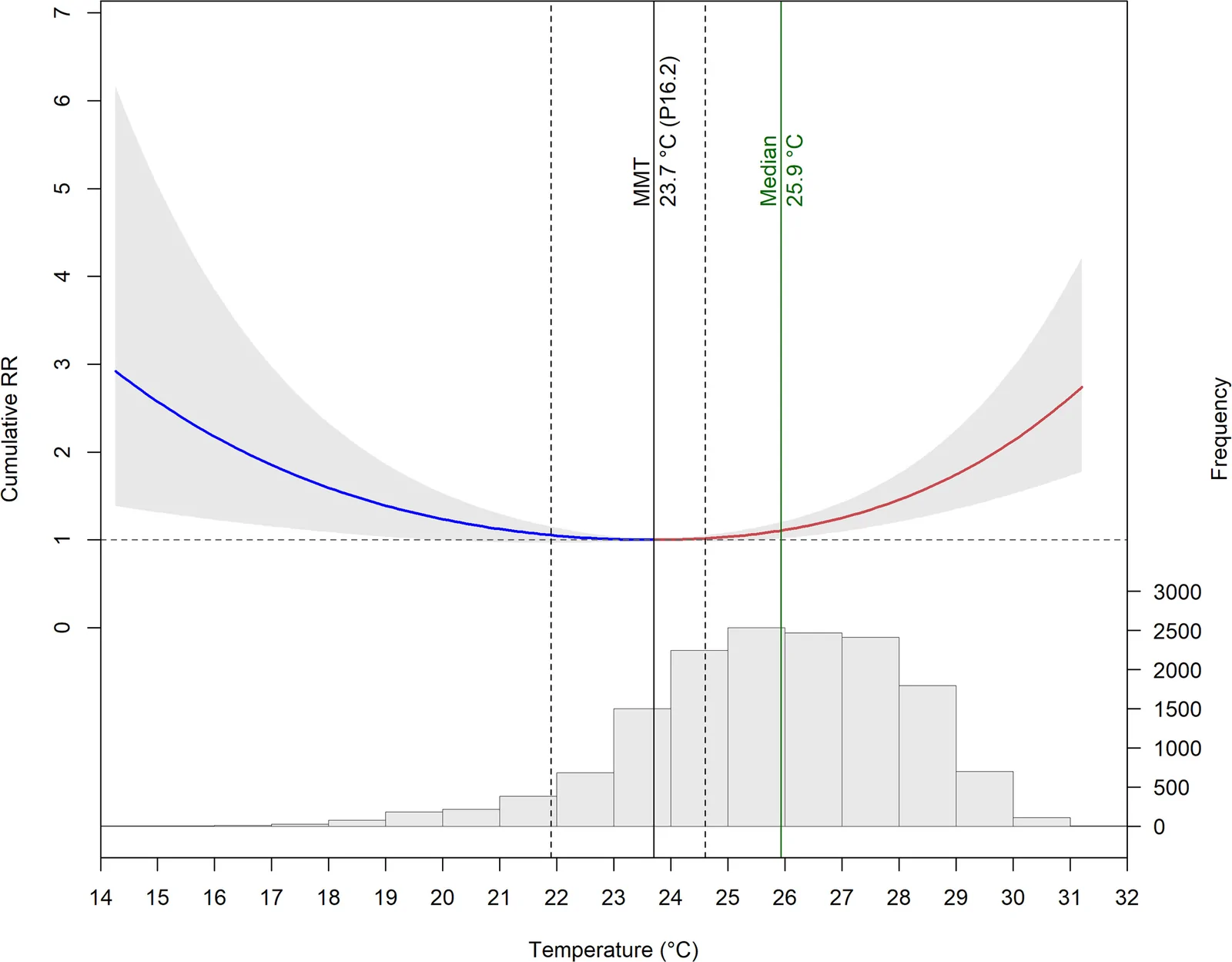
Cumulative exposure-response function of mean temperature and daily piglet mortality over lags 0–7. The blue solid line is the cold effect (temperature below the MMT) and the red solid line shows the heat effect (temperature above the MMT), while the gray area shows the 95% CI. The horizontal dashed line indicates RR = 1. The black vertical solid line shows the MMT, while the vertical dashed lines show the 95% CI of the MMT, and the green vertical solid line shows the median temperature in the study period. The histogram shows the distribution of daily mean temperature across farm-day observations from the 14 farms during the three-year study period.

Below the MMT, estimates showed wide uncertainty until the extreme cold tail, whereas above the MMT, cumulative relative risk increased steadily and accelerated toward the upper tail, consistent with progressively greater mortality risk under higher heat exposure. Because observations were sparse at the extremes of the temperature distribution, the corresponding edge estimates should be interpreted cautiously. At more stable points of the exposure distribution, cumulative RRs (95% CIs) were 1.36 (1.03–1.79) at P1 (19.2 °C), 1.07 (0.97–1.18) at P5 (21.7 °C), 1.76 (1.36–2.28) at P95 (29.0 °C), and 2.08 (1.50–2.87) at P99 (29.9 °C). The exposure histogram indicates that most farm-days occurred above the MMT, approximately the 16th percentile, aligning the higher frequency of exposure with the portion of the curve where risk increases.

The lag-specific temperature effects suggest predominantly acute temperature-related mortality, with the largest relative risks occurring on the day of exposure at both the cold and hot tails of the temperature distribution. As shown in Fig. 4, relative risks remained close to the null across the 0–7-day lag window at moderate temperatures, whereas clearer departures from the null were observed at the extremes. At high temperatures, particularly from P90 upward, the association was strongest at lag 0. At 31.2 °C, the maximum observed mean temperature, the relative risk peaked on the day of exposure (lag 0; RR = 1.49; 95% CI: 1.06–2.09), remained slightly elevated at lag 1, attenuated toward the null over subsequent days, and showed a modest late rebound around lag 7. This later feature was smaller and less precise than the lag-0 association, and its timing and magnitude varied across alternative maximum-lag specifications (Supplementary Figure S2).

**Fig. 4:**
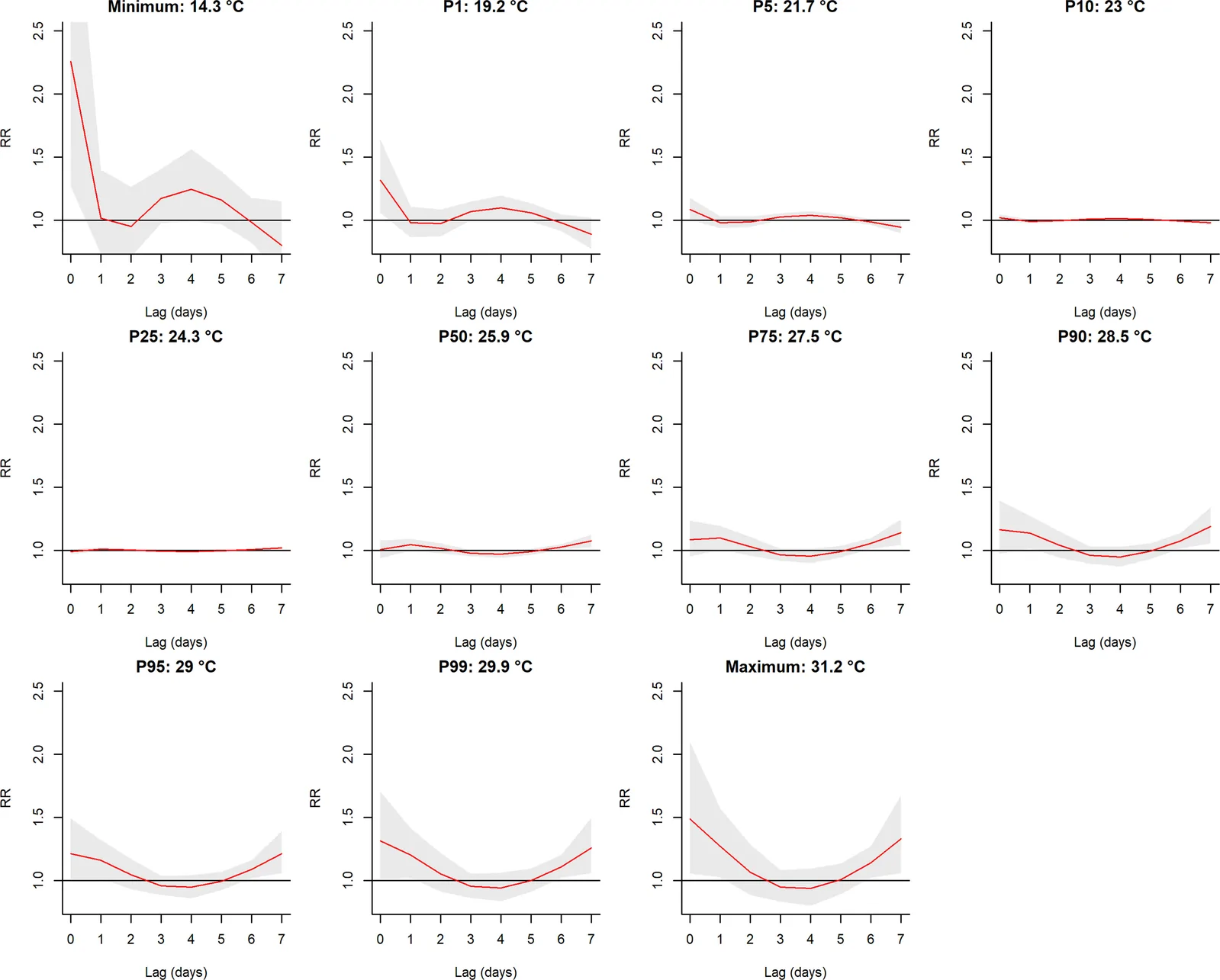
Lag-specific relative risks of piglet mortality at the minimum, P1, P5, P10, P25, P50, P75, P90, P95, P99, and maximum observed mean temperatures (14.3, 19.2, 21.7, 23.0, 24.3, 25.9, 27.5, 28.5, 29.0, 29.9, and 31.2 °C, respectively). P1–P99 denote percentiles of the farm-day distribution of daily mean temperature. Red solid lines show the relative-risk estimates, gray bands show the corresponding 95% confidence intervals, and the horizontal reference line indicates RR = 1. All estimates are centered at the model-derived MMT of 23.7 °C.

At the cold tail, the minimum observed mean temperature of 14.3 °C also showed a marked increase in risk on the day of exposure (lag 0; RR = 2.26; 95% CI: 1.27–4.02), followed by rapid attenuation toward the null at lags 1–2. A smaller elevation was visible around lags 3–5, but confidence intervals were wide, providing limited evidence for a consistent delayed cold effect.

The estimated temperature-attributable burden is summarized in Table 2. The overall attributable fraction (AF) was 16.6% (95% empirical CI [eCI] 9.0–23.0), corresponding to 2916 deaths (95% eCI 1592–4041). This burden was almost entirely attributable to heat (AF 15.5%; 95% eCI 7.7–22.3), whereas cold contributed a small and imprecise fraction (AF 1.1%; 95% eCI −0.2–2.1). Extreme-temperature exposures made a modest contribution: temperatures at or above P99 of the farm-day temperature distribution accounted for 0.7% (95% eCI 0.3–0.9), while temperatures at or below P1 contributed 0.4% (95% eCI 0.0–0.6). Overall, the attributable burden was driven mainly by heat, with limited evidence for a consistent cold-attributable contribution.

**Table 2:** Estimated piglet mortality burden attributable to heat, cold, and temperature extremes.

Effect	Attributable fraction (%)	Attributable number
Overall	16.6 (9.0–23.0)	2916 (1592–4041)
Heat	15.5 (7.7–22.3)	2732 (1353–3924)
Cold	1.1 (−0.2–2.1)	187 (−28–369)
Extreme heat (≥P99)	0.7 (0.3–0.9)	117 (61–161)
Extreme cold (≤P1)	0.4 (0.0–0.6)	66 (7–111)

Values are point estimates with 95% empirical confidence intervals. The model-derived MMT of 23.7 °C was held fixed during coefficient simulation; these intervals are therefore conditional on the estimated MMT. Results propagating MMT uncertainty are presented in Supplementary Table S3. P1 and P99 denote the 1st and 99th percentiles of the farm-day distribution of daily mean temperature, respectively.

In an additional sensitivity analysis, propagating uncertainty in the MMT produced modest changes in the interval limits but did not alter the predominantly heat-related interpretation of the estimated attributable burden (Supplementary Table S3).

In sensitivity analyses using maximum lags of 3, 5, 10, 14, and 21 days, the fitted cumulative association remained non-linear and qualitatively heat-dominated (Supplementary Figure S1). The estimated MMT ranged from 22.8 to 24.6 °C, compared with 23.7 °C in the main 7-day model. Cumulative RRs at P95 ranged from 1.5 to 2.4, whereas estimates at P5 remained close to the null (1.0–1.1). Overall attributable fractions ranged from 10.3% to 24.9% and remained predominantly heat-related (8.6%–24.5%); cold-attributable fractions remained small (0.5%–1.8%) (Supplementary Table S1). Thus, the qualitative heat-dominated pattern was consistent, although the magnitude of the cumulative heat association and attributable burden increased with longer lag windows. The dominant lag-0 elevation was also present across specifications, whereas the timing and magnitude of the smaller later-lag pattern varied (Supplementary Figure S2).

Two additional sensitivity analyses assessed farm-level influence and the impact of including the reconstructed population at risk as an offset. In the leave-one-farm-out analysis, omitting any single farm did not reverse the heat-related association or materially alter its overall shape (Supplementary Figure S3). In a paired sensitivity analysis restricted to 2024, inclusion of the reconstructed population at risk as an offset yielded broadly similar lag-specific patterns but attenuated the lag-0 estimates at P95 and P99 (Supplementary Table S4 and Figure S4).

## Discussion

Across commercial swine farms in Cuba, daily mean temperature exhibited a clear non-linear, lagged association with piglet mortality. The exposure–response function was non-linear and strongly heat-dominated with a marked increase in risk at higher temperatures and a less precise increase at the extreme cold tail. The lag structure indicated that temperature-related mortality was largely acute, concentrating at very short lags and attenuating quickly thereafter. Consistent with this pattern, the estimated temperature-attributable burden was substantial and predominantly heat-related, whereas the cold-attributable component was small and imprecisely estimated. Across alternative maximum-lag specifications, this predominance of heat persisted, but the magnitude of the cumulative association and estimated attributable burden varied appreciably (Supplementary Table S1). Although exposure to temperatures at or above P99 was infrequent in the farm-day temperature distribution, it still contributed a measurable burden, supporting the relevance of both “everyday” warm conditions and short-lived extremes.

### Thermal vulnerability

Our heat-dominated signal aligns with a broad literature on swine physiology showing that pigs are highly vulnerable to heat loads because they have limited functional sweating capacity and rely heavily on respiratory evaporative heat loss and behavioral strategies to maintain thermal balance (Gourdine et al., 2021; Mayorga et al., 2018). When high temperature coincides with high relative humidity, evaporative heat dissipation may be constrained, exacerbating heat strain Godyn et al. (2020); Huynh et al. (2007). Heat stress can reduce feed intake and impair growth in pigs (Liu et al., 2022; Renaudeau, Gourdine & St-Pierre, 2011) and, around farrowing and early lactation, alter sow physiology and postural behavior, with potential consequences for offspring performance (Muns et al., 2016).

Prior studies in temperate indoor and outdoor systems indicate that associations between thermal conditions and piglet outcomes vary with housing and production context (Schild et al., 2019; Wegner et al., 2014). Because the studied Cuban farms used open or semi-open, naturally ventilated maternity buildings with limited active microclimate control, ambient conditions are likely to be a relevant external driver of the maternity-room environment.

Heat-related changes in sow posture could plausibly affect crushing risk or suckling opportunities, but these pathways could not be evaluated because behavioral observations, necropsy data, and cause-specific mortality were unavailable. Trauma/crushing and starvation are among the main proximate causes of pre-weaning mortality (Kielland et al., 2018).

### Lag structure and asymmetry of heat and cold effects

The concentration of elevated risk at lag 0 is consistent with an acute association. Rapid thermoregulatory strain in pigs (Mayorga et al., 2018) and heat-related changes in sow behavior around farrowing (Muns et al., 2016) are plausible contributors, although the available data do not directly identify the underlying mechanisms.

Beyond the immediate lag-0 response, a modest increase in relative risk was visible at lags 6–7 at the upper temperature tail. However, this increase was smaller than the lag-0 effect and was estimated with wider uncertainty. Sensitivity analyses showed that the timing and magnitude of this secondary pattern varied across maximum-lag specifications, whereas the immediate lag-0 elevation was more consistent. Because lag-specific patterns may be sensitive to the selected maximum lag and imposed lag structure, and because unmeasured time-varying farm processes cannot be excluded, the late-lag feature should be regarded as model-sensitive and hypothesis-generating rather than as evidence of a distinct delayed biological pathway (Gasparrini & Armstrong, 2010; Gasparrini, Armstrong & Kenward, 2010).

By contrast, although the cold-attributable burden was small and imprecisely estimated, the lag-specific results suggested an acute increase in risk at the coldest observed temperature. In tropical/subtropical settings like Cuba, cold exposure days are relatively infrequent, which may explain why the overall cold-attributable fraction remained close to zero despite the elevated lag-0 estimate at the extreme lower tail. Neonatal piglets are intrinsically susceptible to hypothermia in the immediate postnatal period, and short-term exposure to low temperatures can rapidly impair mobility, suckling ability, and early survival. Management actions that improve early thermoregulation, such as prompt drying, ensuring access to creep areas, minimizing drafts, and maintaining dry bedding or nesting material where available, can improve early-life thermal stability, although population-level impacts may vary by baseline risk and farm practices (Vande Pol et al., 2021). Therefore, the cold signal in this study is best interpreted as an imprecisely estimated acute association confined to rare, extreme low-temperature conditions, rather than as evidence of a sustained delayed cold-related burden.

### Implications for prevention and climate resilience

A key practical implication is that most of the estimated burden was associated with temperatures above the minimum-mortality temperature, rather than solely with the most extreme hot days. Although exposure to temperatures at or above P99 contributed a measurable burden, the majority of heat-attributable deaths occurred on non-extreme warm days above the MMT. This pattern indicates that mitigation strategies should not be limited to “heatwave-only” responses, but should also target routine warm conditions through improved ventilation, air movement, and accessible cooling at the sow level. A large body of work supports environmental modifications such as evaporative cooling, fogging/misting, sprinkling, increased air velocity, and conductive floor cooling as effective approaches to alleviate heat stress in pig facilities (Godyn et al., 2020). Because the performance of evaporative systems depends on ambient humidity and air movement, combined strategies (e.g., airflow enhancement, shading, conductive cooling) can be particularly valuable in humid tropical settings (Godyn et al., 2020; Huynh et al., 2007). Complementary management actions, including provision of cooled or chilled drinking water, have demonstrated benefits for lactating sows and their litters under high ambient temperatures (Jeon et al., 2006). Recent survey evidence also indicates substantial heterogeneity in perceived heat-stress thresholds and mitigation practices across producers, highlighting opportunities for standardization and context-appropriate guidance (Johnson & Stewart, 2025). In resource-constrained settings, feasible heat-mitigation options include ventilation optimization, shade, water access, targeted cooling during the hottest hours, and farrowing-room microclimate management.

Looking forward, climate change is expected to increase the frequency and intensity of heat conditions relevant to pig production, with growing importance of adaptation and potential productivity impacts (Niu, Zhong & Hu, 2024; Renaudeau & Dourmad, 2022). Our results indicate that the estimated temperature-attributable burden was predominantly heat-related in the studied Cuban farms, underscoring the need to integrate heat risk into routine herd health planning and climate-resilience strategies.

### Strengths, limitations, and future work

This study is strengthened by the space–time stratified case-crossover design and the DLNM specification, which together support robust estimation of non-linear and delayed temperature effects while controlling for time-invariant farm characteristics, seasonality, long-term temporal trends, and day-of-week patterns. Further strengths include the size and geographic scope of the dataset—17,599 recorded deaths over three years from 14 commercial farms across 12 Cuban municipalities—and the generation of multi-farm evidence under tropical production conditions, where comparable epidemiological information is limited. Applying this framework to pre-weaning piglet mortality and estimating attributable fractions and numbers—including separate model-based burden estimates for routine warm conditions and rare extreme-heat events—strengthen its methodological and practical relevance for temperature-risk management in Cuban swine production.

A complete daily denominator for the number of suckling piglets at risk was not available for the full 2022–2024 period. Accordingly, the reported RRs represent model-estimated relative changes in daily recorded pre-weaning death counts within farms rather than mortality-rate ratios based on an exact daily population at risk, while the AF and AN quantify the corresponding model-based burden among the recorded deaths. The space–time stratified design reduces confounding by stable farm characteristics, seasonality, long-term trends, and day-of-week patterns. Nevertheless, unmeasured short-term variation in the number or age structure of piglets within farrowing units may have influenced the magnitude of the estimated associations and model-derived attributable burden.

Limitations include potential exposure misclassification because municipality-level ambient temperature and relative humidity may not fully represent the thermal microclimate within individual maternity rooms or farrowing crates/cubicles, which may affect the estimated associations. Nevertheless, because the study farms used open or semi-open naturally ventilated maternity buildings with limited active microclimate control, ambient conditions are likely to be a relevant external driver of the thermal environment in this production context. Accordingly, the estimated MMT should be interpreted as the minimum of the fitted association on the municipality-level ambient temperature scale used in this study, rather than as a precise pen-level biological threshold.

Because these administrative mortality records were collected for routine production and animal-health monitoring, they lacked cause-specific and necropsy information, direct behavioral observations, and time-varying data on housing, management, mitigation practices, and infectious disease events. This limited mechanistic interpretation and the assessment of effect modification. We could not formally assess whether mortality-recording practices differed across farms or changed during the study period.

The observational design does not establish causality, and the daily resolution of exposure and mortality means that lag 0 represents a same-day association without resolving within-day temporal ordering. Caution is also warranted when generalizing the findings beyond the studied Cuban farms, particularly to production systems with different climatic, housing, or management conditions.

Further work should incorporate prospective collection of daily population-at-risk denominators, cause-specific mortality, and indoor temperature/humidity or heat indices where available. Future studies should also evaluate effect modification by maternity-building characteristics, crate/cubicle design, ventilation and cooling practices, bedding or curtain use, and other management adaptations, as well as develop scenario-based projections to quantify avoidable mortality under feasible heat-mitigation packages.

## Conclusions

In the Cuban commercial swine farms studied during 2022–2024, municipality-level daily mean ambient temperature was non-linearly associated with daily recorded counts of pre-weaning piglet deaths, with a predominantly acute lag pattern. The model-derived minimum-mortality temperature was 23.7 °C, and the fitted relative risk increased progressively at warmer temperatures, with the largest excess risks concentrated within the first few days after exposure.

The estimated temperature-attributable burden under the fitted model was predominantly heat-related, whereas the cold-attributable component was small and imprecisely estimated. Although extreme heat contributed a measurable fraction of mortality, most heat-attributable deaths occurred on non-extreme warm days above the MMT, highlighting routine heat conditions as key targets for prevention.

These findings support prioritizing heat abatement and microclimate management in farrowing environments, alongside preparedness for hot periods, as core components of climate-resilient swine production in Cuba. Future studies incorporating exact daily piglet denominators, indoor microclimate data and farm-level mitigation practices should refine exposure assessment and identify the most effective intervention packages to reduce temperature-attributable piglet mortality.

## Ethics declaration

Ethics committee approval was not required. Ethical approval was not required. The study involved secondary analysis of routinely collected production and mortality records and meteorological reanalysis data. Formal authorization for the secondary use of the farm records was obtained from GENSPOR. No animal experimentation or additional procedures beyond standard farm practice were performed.

### Ethics approval

Formal authorization for the secondary use of the farm records was obtained from the Cuban Porcine Genetic Company (GENSPOR), which provided the data under the condition that they would not be shared or made publicly available. The study was based on secondary analysis of routinely collected production/mortality records and meteorological reanalysis data, and did not involve animal experimentation or any additional procedures on animals beyond standard farm practice.

## Declaration of generative AI and AI-assisted technologies in the writing process

During the preparation of this manuscript, the authors used generative AI and AI-assisted technologies (ChatGPT, OpenAI) to support language editing and improve clarity and readability of the text. The authors reviewed and edited all AI-assisted content and take full responsibility for the integrity, accuracy, and originality of the manuscript.

## Financial support statement

Osvaldo Fonseca Rodríguez is supported by the Ramón y Cajal Programme (grant RYC2022-037029-I), funded by the Ministerio de Ciencia, Innovación y Universidades and the Agencia Estatal de Investigación (MICIU/AEI/10.13039/501100011033), and by the European Social Fund Plus (ESF+).

IRTA is supported by the CERCA Programme/Generalitat de Catalunya.

## CRediT authorship contribution statement

**O. Fonseca-Rodríguez:** Writing – review & editing, Writing – original draft, Visualization, Supervision, Methodology, Investigation, Formal analysis, Data curation, Conceptualization. **E.A. Curbelo-Benítez:** Writing – review & editing, Writing – original draft, Formal analysis, Data curation. **F.A. Morales-Calvo:** Writing – review & editing, Writing – original draft, Formal analysis. **L. Pailler-García:** Writing – review & editing, Formal analysis, Data curation. **M.E. Licourt-Hernández:** Writing – review & editing, Data curation. **O. Uffo-Reinosa:** Writing – review & editing, Data curation. **P. Alfonso:** Writing – original draft, Supervision, Data curation. **D. de las Nieves Montano-Valle:** Writing – review & editing, Writing – original draft, Supervision, Data curation, Conceptualization.

## Declaration of competing interest

The authors declare that they have no known competing financial interests or personal relationships that could have appeared to influence the work reported in this paper.

## Data Availability

The farm-level daily piglet mortality records analyzed in this study cannot be publicly shared under the conditions established by the data owner. The ERA5-Land meteorological inputs are publicly available through the Copernicus Climate Change Service Climate Data Store (Muñoz Sabater, 2019). The supplementary reproducibility package includes two separate R Markdown files, one for generating a fully synthetic farm-day dataset and one for applying the analytical workflow, together with a validated reference synthetic dataset and an accompanying README. The synthetic dataset contains no actual farm or location identifiers or empirical mortality records and permits reproduction of the analytical workflow and the types of tables and figures presented, but not the exact numerical results reported for the confidential data.
