# Supplementary material for: Nonlinear and lagged association between ambient temperature and pre-weaning piglet mortality in Cuba
Source: Vet Anim Sci. 2026 Oct 22;34:100909. doi: 10.1016/j.vas.2026.100909 (PMC13735290; doi:10.1016/j.vas.2026.100909)
Supplement: Supplementary file 1 [file mmc1.docx]

**Supplementary material**

**Nonlinear and lagged association between ambient temperature and pre-weaning piglet mortality in Cuba**

O. Fonseca-Rodríguez ^a,b^, E. A. Curbelo-Benítez ^c^, F. A. Morales-Calvo ^a,b^, L. Pailler-García ^a,b^, M. E. Licourt-Hernández ^c^, O. Uffo-Reinosa ^d^, P. Alfonso ^c^, D. de las Nieves Montano-Valle ^c^

*^a^ Unitat mixta d’Investigació IRTA-UAB en Sanitat Animal. Centre de Recerca en Sanitat Animal (CReSA). Campus de la Universitat Autònoma de Barcelona (UAB), Bellaterra, 08193, Catalonia. Spain.*

*^b^ IRTA. Programa de Sanitat Animal. Centre de Recerca en Sanitat Animal (CReSA). Campus de la Universitat Autònoma de Barcelona (UAB), Bellaterra, 08193, Catalonia. Spain.*

*^c^ Epidemiology Group, National Center for Animal and Plant Health (CENSA), World Organization for Animal Health (WOAH) Collaborating Center for the Reduction of the Risk of Disaster in Animal Health, San José de las Lajas, Cuba.*

*^d^ Centro Nacional de Sanidad Agropecuaria (CENSA), Laboratorio de Ensayo para el Control de la Calidad de los Alimentos (CENLAC), San José de las Lajas, Cuba.*

**Corresponding author:** Osvaldo Fonseca Rodríguez.

**Email:** osvaldo.fonseca@irta.cat

**Table S1.** Sensitivity of key model estimates to alternative maximum lags (3, 5, 7, 10, 14, and 21 days).

| **Model** | **Maximum lag (days)** | **MMT (°C)** | **Cumulative RR at minimum** | **Cumulative RR at P5** | **Cumulative RR at P95** | **Cumulative RR at maximum** | **AF overall (%)** | **AF heat (%)** | **AF cold (%)** |
| --- | --- | --- | --- | --- | --- | --- | --- | --- | --- |
| Lag 3 | 3 | 24.6 | 4.0 (2.2–7.3) | 1.1 (1.1–1.2) | 1.5 (1.2–1.8) | 2.1 (1.5–3.0) | 10.3 | 8.6 | 1.8 |
| Lag 5 | 5 | 24.4 | 3.5 (1.8–6.7) | 1.1 (1.0–1.2) | 1.5 (1.2–1.9) | 2.2 (1.5–3.2) | 11.5 | 10.0 | 1.5 |
| Lag 7 (main model) | 7 | 23.7 | 2.9 (1.4–6.2) | 1.1 (1.0–1.2) | 1.8 (1.4–2.3) | 2.7 (1.8–4.2) | 16.6 | 15.5 | 1.1 |
| Lag 10 | 10 | 23.4 | 3.1 (1.3–7.3) | 1.1 (0.9–1.2) | 2.0 (1.5–2.7) | 3.5 (2.2–5.6) | 20.6 | 19.6 | 1.0 |
| Lag 14 | 14 | 22.8 | 2.5 (0.9–6.7) | 1.0 (0.9–1.2) | 2.3 (1.7–3.2) | 4.2 (2.4–7.1) | 24.0 | 23.5 | 0.5 |
| Lag 21 | 21 | 22.8 | 2.6 (0.8–8.6) | 1.0 (0.9–1.2) | 2.4 (1.6–3.5) | 4.4 (2.3–8.3) | 24.9 | 24.5 | 0.5 |

*Abbreviations: AF, attributable fraction; MMT, minimum-mortality temperature; RR, relative risk. Values in parentheses are 95% confidence intervals for cumulative RRs. P5 and P95 denote the 5th and 95th percentiles of the farm-day distribution of daily mean temperature, respectively. Minimum and maximum refer to the observed temperature extremes. Cumulative RRs and AF estimates were calculated using the MMT from the main 7-day model (23.7 °C) as a common reference across maximum-lag specifications; the MMT column reports the minimum estimated separately for each specification. qAIC values were not compared across maximum-lag specifications because their complete lag-history samples differed.*

**Table S2.** qAIC comparison of all 27 candidate smoothing specifications for the main 0-7-day distributed lag nonlinear model.

| **Rank** | **Temperature df** | **Lag knots** | **Humidity df** | **Number of parameters (K)** | **qAIC** | **Delta qAIC** | **Selected** |
| --- | --- | --- | --- | --- | --- | --- | --- |
| 1 | 2 | 3 | 3 | 14 | 14,364.02 | 0.00 |  |
| **2** | **2** | **2** | **3** | **12** | **14,365.58** | **1.56** | **Yes** |
| 3 | 2 | 3 | 4 | 15 | 14,365.74 | 1.73 |  |
| 4 | 2 | 4 | 3 | 16 | 14,366.41 | 2.39 |  |
| 5 | 2 | 3 | 2 | 13 | 14,366.91 | 2.89 |  |
| 6 | 2 | 2 | 4 | 13 | 14,367.30 | 3.28 |  |
| 7 | 2 | 4 | 4 | 17 | 14,368.14 | 4.12 |  |
| 8 | 2 | 2 | 2 | 11 | 14,368.39 | 4.37 |  |
| 9 | 2 | 4 | 2 | 15 | 14,369.30 | 5.28 |  |
| 10 | 3 | 3 | 3 | 19 | 14,371.06 | 7.05 |  |
| 11 | 3 | 2 | 3 | 16 | 14,371.12 | 7.11 |  |
| 12 | 4 | 2 | 3 | 20 | 14,372.13 | 8.11 |  |
| 13 | 3 | 3 | 4 | 20 | 14,372.81 | 8.80 |  |
| 14 | 3 | 2 | 4 | 17 | 14,372.86 | 8.85 |  |
| 15 | 4 | 2 | 2 | 19 | 14,372.93 | 8.91 |  |
| 16 | 3 | 3 | 2 | 18 | 14,373.00 | 8.98 |  |
| 17 | 3 | 2 | 2 | 15 | 14,373.07 | 9.06 |  |
| 18 | 4 | 2 | 4 | 21 | 14,373.93 | 9.91 |  |
| 19 | 3 | 4 | 3 | 22 | 14,374.01 | 9.99 |  |
| 20 | 4 | 3 | 3 | 24 | 14,374.15 | 10.13 |  |
| 21 | 4 | 3 | 2 | 23 | 14,374.93 | 10.91 |  |
| 22 | 3 | 4 | 4 | 23 | 14,375.75 | 11.74 |  |
| 23 | 4 | 3 | 4 | 25 | 14,375.95 | 11.94 |  |
| 24 | 3 | 4 | 2 | 21 | 14,376.05 | 12.03 |  |
| 25 | 4 | 4 | 3 | 28 | 14,377.86 | 13.84 |  |
| 26 | 4 | 4 | 2 | 27 | 14,378.76 | 14.74 |  |
| 27 | 4 | 4 | 4 | 29 | 14,379.66 | 15.64 |  |

*All 27 candidates used a 0-7-day lag window, natural cubic splines for temperature and relative humidity, and a natural cubic spline for lag with internal knots equally spaced on the log-lag scale. qAIC = -2 log(L)/c-hat + 2K, using a common c-hat of 2.624 estimated from the global candidate (temperature df = 4, lag knots = 4, humidity df = 4). K denotes the number of non-eliminated regression coefficients plus one parameter for dispersion; eliminated stratum effects were not counted. Delta qAIC is relative to the minimum. The selected model is the candidate with the lowest K among models with Delta qAIC < 2; ties were resolved by the lower qAIC. All candidates used 10,512 farm-days.*

**Figure S1.** Cumulative temperature-mortality association under alternative maximum-lag specifications. The vertical dashed line indicates the MMT estimated from the main 7-day model (23.7 °C), shown as a common reference across specifications. The horizontal dashed line indicates RR = 1.

**Figure S2.** Lag-response curves at selected temperature values (minimum, P1, P5, P95, P99, and maximum) under alternative maximum-lag specifications. Rows represent the maximum-lag specification and columns represent the selected temperature value. Red lines show relative-risk estimates, gray bands show 95% confidence intervals, and the horizontal dashed line indicates RR = 1. All estimates were centered at the minimum-mortality temperature (MMT) from the main 7-day model (23.7 °C), used as a common reference across specifications.

**Figure S3.** Leave-one-farm-out influence analysis of the temperature–mortality association. Blue curves and shaded bands show the estimates and corresponding 95% confidence intervals from the full-data model; gray curves show estimates from 14 models, each omitting one farm. The upper panel presents cumulative relative risks over lags 0–7, and the lower panel presents lag-0 relative risks. All models used the same temperature–lag basis specification and prediction grid and were centered at the full-data minimum-mortality temperature (MMT) of 23.7 °C. The dotted vertical line indicates the MMT, and the dashed horizontal lines indicate RR = 1.

Table S3. Comparison of uncertainty intervals for the estimated temperature-attributable burden with the minimum-mortality temperature fixed at 23.7 °C and with MMT uncertainty propagated.

| **Component** | **Measure** | **Point estimate** | **Fixed-MMT 95% interval** | **MMT-propagated 95% interval** |
| --- | --- | --- | --- | --- |
| Overall | AF (%) | 16.6 | 9.0–23.0 | 9.9–24.4 |
| Overall | AN | 2,916 | 1,592–4,041 | 1,748–4,291 |
| Heat | AF (%) | 15.5 | 7.7–22.3 | 8.6–23.7 |
| Heat | AN | 2,732 | 1,353–3,924 | 1,507–4,179 |
| Cold | AF (%) | 1.1 | -0.2–2.1 | 0.2–2.4 |
| Cold | AN | 187 | -28–369 | 37–414 |
| *Abbreviations: AF, attributable fraction; AN, attributable number; MMT, minimum-mortality temperature. Point estimates and fixed-MMT intervals use the model-derived MMT of 23.7 °C. MMT-propagated intervals were obtained from 10,000 joint coefficient simulations, with the MMT re-estimated for each draw. The median simulated MMT was 23.7 °C (95% CI: 21.9–24.6 °C); 0.04% of simulated MMTs occurred at a boundary of the observed temperature range.* | | | | |

**Reconstructed population-at-risk sensitivity analysis for 2024**

Daily birth records required to reconstruct the population at risk were available only for 2024. For each farm, the daily population at risk was reconstructed by adding recorded births to age-specific cohorts of suckling piglets. The risk set was calculated before subtracting that day’s recorded deaths; deaths were subsequently removed proportionally from the surviving cohorts, and piglets were assumed to leave the risk set through weaning uniformly between 26 and 33 days of age. The first 33 days of 2024 were excluded as a wash-in period to allow reconstruction of the prevailing cohorts. Otherwise identical models, with and without the logarithm of the reconstructed population included as an offset, were fitted to the same 3,424 farm-days, comprising 5,136 deaths from 14 farms.

**Table S4.** Lag-0 relative risks from the paired 2024 offset sensitivity analysis

| Model specification | MMT (°C) | P01: 19.6°C | P05: 21.4°C | P95: 29.2°C | P99: 29.9°C |
| --- | --- | --- | --- | --- | --- |
| Without offset | 24.1 | 1.17  (0.77–1.77) | 1.05  (0.85–1.28) | 1.77  (1.23–2.56) | 2.02  (1.30–3.14) |
| With reconstructed-population offset | 24.4 | 1.18  (0.78–1.80) | 1.06  (0.86–1.30) | 1.52  (1.05–2.20) | 1.68  (1.08–2.62) |

*Values are lag-0 relative risks (95% confidence intervals), centered at the common reference temperature of 23.7°C. MMT was identified within the P01–P99 temperature range. Both models were fitted to the same 3,424 farm-days, comprising 5,136 deaths from 14 farms.*

***Figure S4.*** *Comparison of lag-specific relative risks with and without a reconstructed-population offset in the 2024 sensitivity analysis. Relative risks are presented at the 1st (P01), 5th (P05), 95th (P95), and 99th (P99) percentiles of daily mean temperature and are centered at 23.7°C. Both models were fitted to the same 3,424 farm-days from 14 farms. Shaded areas represent 95% confidence intervals, and the dashed horizontal line indicates a relative risk of 1.*
